# Metastasis to lateral lymph nodes with no mesenteric lymph node involvement in low rectal cancer: a retrospective case series

**DOI:** 10.1186/s12957-020-02068-3

**Published:** 2020-11-06

**Authors:** Peng Li, Zhichun Zhang, Yuanda Zhou, Qingsheng Zeng, Xipeng Zhang, Yi Sun

**Affiliations:** Department of Colorectal Surgery, Tianjin Union Medical Center, Tianjin, 300000 China

## Abstract

**Purpose:**

The aim of this study is to examine the pattern of lymph node metastasis (lateral vs. mesenteric lymph nodes) in low rectal cancer.

**Methods:**

This retrospective analysis included all patients undergoing laparoscopic total mesorectal excision plus lateral lymph node dissection for advanced low rectal cancer (up to 8 cm from the anal verge) during a period from July 1, 2017, to August 31, 2019, at the Department of Colorectal Surgery, Tianjin Union Medical Center. The decision to conduct lateral lymph node dissection was based on positive findings in preoperative imaging assessments.

**Results:**

A total of 42 patients were included in data analysis. Surgery was successfully completed as planned, without conversion to open surgery in any case. A minimum of 10 mesenteric lymph nodes and 1 lateral lymph node on each side were dissected in all patients. Pathologic examination of resected specimens showed no metastasis to either mesenteric or lateral lymph nodes in 7 (16.7%) case, metastasis to both mesenteric and lateral lymph nodes in 26 (61.9%) cases, metastasis to mesenteric but not lateral lymph nodes in 4 (9.5%) cases, and metastasis to lateral but not mesenteric lymph nodes in 5 (11.9%) cases (*n* = 2 in the obturator region; *n* = 3 in the iliac artery region).

**Conclusion:**

A clinically significant proportion of low rectal cancer patients have metastasis to lateral lymph nodes without involvement of mesenteric lymph nodes. More carefully planned prospective studies are needed to verify this preliminary finding.

## Introduction

In patients with low rectal cancer (up to 8 cm from the anal verge), estimated rate of lateral lymph node metastasis is 16–23% [1]. The most recent Japanese Society for Cancer of the Colon and Rectum (JSCCR) Guidelines for the treatment of colorectal cancer classify metastasis to lateral lymph nodes as local metastasis and recommend lateral lymph node dissection (LLND) in both stage II and III low rectal cancers [2]. The NCCN Guidelines recommend chemoradiotherapy (CRT) plus total mesorectal excision (TME) treatment for lateral lymph node metastasis [3]. A recent study reported 19.5% 5-year local recurrence rate after CRT plus TME versus 5.5% 5-year local recurrence rate after CRT plus TME and LLND in patients with lateral lymph nodes at least 7 mm in diameter, supporting the notion that lateral lymph node involvement represents local metastasis [4].

In this retrospective analysis, we examined the metastasis profile (lateral vs. mesenteric lymph nodes) in a group of low rectal cancer patients with suspected lateral lymph node involvement based on preoperative imaging assessments. The results showed metastasis to lateral but not mesenteric lymph nodes in 5 out of 42 patients, supporting the notion that lateral lymph node metastasis should be regarded as local metastasis.

## Patients and methods

We identified all patients receiving laparoscopic TME plus lateral lymph node dissection for advanced low rectal cancer (up to 8 cm from the anal verge on magnetic resonance imaging (MRI)) during a period from July 1, 2017, to August 31, 2019, at the Department of Colorectal Surgery, Tianjin Union Medical Center.

If the short diameter of the largest lymph node was at least 7 mm in diameter in MRI or the rectal lesions accord with the standard of neoadjuvant CRT therapy evaluated by a multidisciplinary team, neoadjuvant CRT was recommended to the patients. In long-course radiotherapy, a total dose of 45–50 Gy was given over 5 weeks. Typical chemotherapy regimen was CapeOX: two cycles of an intravenous oxaliplatin (130 mg/m^2^ per day) for 1 day and oral capecitabine (1000 mg/m^2^ twice per day) for 14 days in the first and fourth weeks of radiotherapy. The short diameter of the largest lymph was re-evaluated after neoadjuvant therapy. If the preoperative short diameter of the largest lymph node was at least 5 mm, TME + LLND was performed 6–8 weeks after neoadjuvant CRT. If the patient refused to receive neoadjuvant CRT, TME + LLND was performed immediately.

TME and lateral lymph node dissection were performed using a fascial space priority approach, as previously described [5].

### Statistical analysis

In addition to descriptive statistics, we also compared the demographic and pathologic features among subjects with different metastasis pattern (i.e., metastasis to both mesenteric and lateral, metastasis to mesenteric but not lateral, and metastasis to lateral but not mesenteric lymph nodes). Continuous variables are expressed as mean ± standard deviation if following normal distribution, and as median (range) otherwise. Categorical variables are presented as numbers (%). All analyses were conducted using SPSS Statistics (Version 25.0).

## Results

A total of 42 patients were included in data analysis. Surgery was completed as planned, with no conversion to open surgery. Median distance from the lesion to the anal verge was 4.8 cm (range 0–8) (Table 1). Sixteen patients received neoadjuvant CRT. Twenty-eight patients received unilateral lateral lymph node dissection, and the remaining 14 patients received bilateral dissection. A minimum of 10 mesenteric lymph nodes and 1 lateral lymph node on each side were dissected in all patients.

**Table 1:** Demographic and clinical characteristics

	Entire sample (*n* = 42)	meso+, lat+ (*n* = 26)	meso+, lat− (*n* = 4)	meso−, lat+ (*n* = 5)	meso−, lat− (*n* = 7)
Age (years), mean ± SD	58.79 ± 10.69	58.23 ± 11.14	64.75 ± 12.09	58.80 ± 6.53	57.43 ± 11.66
Male sex, *n* (%)	24 (57.1)	16 (61.5)	2 (50.0)	2 (40.0)	4 (57.1)
BMI (kg/m^2^), mean ± SD	24.61 ± 3.37	24.48 ± 3.37	24.52 ± 5.32	25.56 ± 3.56	24.64 ± 2.72
Distance from AV (cm), median (range)	4.8 (0–8)	4.55 (0–8)	5.65 (1–8)	4.8 (3–5.5)	5.6 (3.5–8)
cT Stage, *n* (%)					
ct2	2 (4.8)				1 (14.3)
cT3	31 (73.8)	21 (80.8)	3 (75.0)	4 (80.0)	4 (57.1)
cT4	9 (21.4)	5 (19.2)	1 (25.0)	1 (20.0)	2 (28.6)
cN Stage, *n* (%)					
cN1	21 (50.0)	10 (38.5)	1 (25)	5 (100)	4 (57.1)
cN2	21 (50.0)	16 (61.5)	3 (75)		3 (42.9)
nCRT, *n* (%)	16 (38.1)	10 (38.5)	1 (25.0)	2 (40)	3 (42.9)

*AV* anal verge, *BMI* body mass index, *lat* lateral lymph nodes, *meso* mesenteric lymph nodes

Metastasis was verified in both mesenteric and lateral lymph nodes in 26 (61.9%) patients, in mesenteric but not in lateral lymph nodes in 4 (9.5%) patients, and in lateral but not mesenteric lymph nodes in 5 (11.9%) patients. In the 5 cases with metastasis to lateral but not mesenteric lymph nodes, involved lymph nodes were in the obturator region in 2 cases, and in the iliac artery region in the remaining 3 cases.

Surgical approach, pathologic staging, and the extent of lymph node dissection in the entire study sample and in patients with different patterns of lymph node metastasis are shown in Table 2. The median follow-up in the 5 patients with lateral but no mesenteric lymph node metastasis was 13 (1–31) months; no recurrence was observed.

**Table 2:** Surgical and pathologic characteristics

	Entire sample (*n* = 42)	meso+, lat+ (*n* = 26)	meso+, lat− (*n* = 4)	meso−, lat+ (*n* = 5)	meso−, lat− (*n* = 7)
Surgical approach, *n* (%)					
Abdominoperineal resection	18	10 (38.5)	2 (50.0)	2 (40)	4 (57.1)
Anterior resection	19	12 (46.2)	1 (25.0)	3 (60)	3 (42.9)
Hartmann’s procedure	5	4 (15.4)	1 (25.0)		
LLND dissection, *n* (%)					
Unilateral	28	13 (50.0)	3 (75.0)	5 (100)	7 (100)
Bilateral	14	13 (50.0)	1 (25.0)		
TNM stage, *n* (%)					
0	1 (2.4)				1 (14.3)
I	1 (2.4)				1 (14.3)
II	4 (9.5)				4 (57.1)
III	35 (83.3)	25 (96.2)	4 (100)	5 (100)	1 (14.3)
IV	1 (2.4)	1 (3.8)			
Mesenteric lymph nodes, M (range)					
No. of dissection per patient	3 (0–23)	17 (10–29)	17.5 (12–20)	16 (12–21)	15 (11–25)
No. of metastasis per patient	16.5 (10–29)	4.5 (1–23)	5 (2–10)	0	0
Lateral lymph nodes, M (range)					
No. of dissection per each side	7.5 (1–41)	6 (1–41)	15 (7–24)	8 (3–13)	8 (4–19)
No. of metastasis per each side	1 (0–10)	1 (0–10)	0	1 (1–2)	0
Metastatic lymph node location, *n* (%)					
Iliac artery region				3 (60)	
Obturator region				2 (40)	

*lat* lateral lymph nodes, *meso* mesenteric lymph nodes, *(−)* negative, *(+)* positive

## Discussion

In a previous study, we reported a fascial space priority approach for lateral lymph node dissection in patients with rectal cancer [5]. Using this approach, we found in the current study that 5 out of a total of 42 patients (11.9%) with low rectal cancer had metastasis to lateral lymph nodes but not to mesenteric lymph nodes. This finding supports managing lateral lymph node involvement as local metastasis [6–8] and suggests the possibility that lateral lymph nodes may be sentinel lymph nodes in some patients.

Lymphatic drainage of the lower rectum passes to external pelvic (inguinal area) or pelvic (iliac vessels and anterior sacral) lymph nodes, or to the root of inferior mesenteric artery along the superior rectal artery. In a study by Akiyoshi and colleagues, prognosis did not differ significantly between patients with N2a and those with lymph node metastasis either in the external iliac artery region (5-year overall survival, 45% vs 45%, *P* = 0.9585; 5-year cancer-specific survival: 51% vs 49%, *P* = 0.5742) or in the internal iliac artery region (5-year overall survival: 32% vs 29%, *P* = 0.3342; 5-year cancer-specific survival: 37% vs 34%, *P* = 0.4347) [9], suggesting that the lateral lymph node involvement should be regarded as local metastasis. The findings from the current study supported such a notion. Lymphatic mapping technology can be adopted to study drainage pattern of low rectal cancer [10].

Few studies have examined the prognosis of patients with lateral lymph node metastasis but no mesenteric lymph node metastasis. Based on a study by Takahashi et al. [6], the 5-year survival rate was 90.1% in patients with no metastasis to either mesenteric or lateral lymph nodes, 75% in patients with metastasis to lateral but not mesenteric lymph nodes, 67.7% in patients with metastasis to mesenteric but not lateral lymph nodes, and 32% in patients with metastasis to both lateral and mesenteric lymph nodes. Akiyoshi and colleagues argued that metastasis to lymph nodes located in the area medial to internal iliac artery should be classified as N2a and those located in the area lateral to internal iliac artery should be classified as N2b [9]. Despite such detailed differences, the prognosis of patients with metastasis to lateral but not mesenteric lymph nodes is clearly better than in patients with metastasis to both lateral and mesenteric lymph nodes. Studies with larger sample size and with a focus on the long-term survival in patients with distinct lymph node metastasis (lateral vs mesenteric) are needed to examine the clinical significance.

Lateral lymph node dissection could influence pathologic staging and hence postoperative management of the patients. In the current study, the 5 patients with metastasis to lateral but not mesenteric lymph nodes would have been classified as pN0 and stage II if lateral lymph nodes were not dissected. With erroneous staging, adjuvant chemotherapy after surgery would not be recommended. In low rectal cancer patients with MRI evidence for lateral lymph node involvement but no metastasis to mesenteric lymph node, CRT should be initiated; in patients who do not respond to CRT, LLND should be conducted. For patients with no mesenteric lymph node metastasis upon pathologic examination (regardless of the lateral lymph node status), the 2020 NCCN Guideline recommends the “watch and wait” approach. The results from the current study suggest that more attention should be given to lateral lymph node metastasis after neoadjuvant chemoradiation [3].

The AJCC colorectal cancer staging Guideline [11] classifies lymph nodes in the iliac artery region as regional, but considers metastasis to lymph nodes in obturator artery region as distant metastasis. Two patients in the current study had metastasis to lymph nodes in the obturator but not iliac artery lymph nodes or mesenteric lymph nodes. Based on this finding, we speculate that obturator lymph nodes should also be regarded as regional. Cirocchi et al. reported that the pooled prevalence estimate of LCA absence was 1.2% (95% CI 0.0**–**3.6%). This rare absence of the left colonic artery/superior rectal artery or variation in lymphatic drainage may also contribute to this phenomenon [12]. Due to very small number of the cases, this speculation must be examined in future studies.

There are several important limitations in the current study. First, this is a retrospective analysis of the patients receiving TME plus LLND for low rectal cancer. Due to the retrospective nature, there were no strict criteria for LLND. Nevertheless, we adopted a general set of indications for LLND. Another important limitation is the use of neoadjuvant CRT in some but not all patients, which may have influenced the pathologic staging. Third, we did not conduct systematic follow-up. As a result, the clinical significance of metastasis to lateral but not mesenteric lymph nodes remains ambiguous. The sample size is also relatively small, and we could not compare the baseline features across patients with different pattern of lymph node metastasis.

## Conclusion

A clinically meaningful proportion of low rectal cancer patients had metastasis to lateral but not mesenteric lymph nodes. The presence of this group of patients indicates a need to re-evaluate whether metastasis to lateral lymph nodes represents local or distant metastasis.

## Data Availability

The dataset is available upon request.
